# The trends and future projections of intraocular foreign bodies among children and adolescents: a global analysis

**DOI:** 10.3389/fmed.2025.1512959

**Published:** 2025-02-13

**Authors:** Zupeng Lu, Xindan Xing, Wen Li, Tong Qiao

**Affiliations:** ^1^Department of Ophthalmology, Shanghai Children’s Hospital, School of Medicine, Shanghai Jiao Tong University, Shanghai, China; ^2^Department of Ophthalmology, Shanghai General Hospital, School of Medicine, Shanghai Jiao Tong University, Shanghai, China; ^3^National Clinical Research Center for Eye Diseases, Shanghai Key Laboratory of Ocular Fundus Diseases, Shanghai, China; ^4^Shanghai Engineering Center for Visual Science and Photomedicine, Shanghai Engineering Center for Precise Diagnosis and Treatment of Eye Diseases, Shanghai, China

**Keywords:** global, intraocular foreign bodies, GBD, Bayesian age-period-cohort, SDI

## Abstract

**Objective:**

The aim was to evaluate the disease burden of intraocular foreign bodies (IOFBs) among children and adolescents globally based on the Global Burden of Disease, Injury, and Risk Factor Study 2021 (GBD 2021).

**Methods:**

Data were extracted from the GBD 2021. The incidence and DALYs number and rate with 95% uncertainty interval (UI) and estimated annual percent change (EAPC) of IOFBs were estimated by age, sex and socio-demographic index (SDI) region. A Bayesian age-period-cohort (BAPC) analysis model was used to predict trends in the next ten years.

**Results:**

Globally, it is estimated that the incidence number of IOFBs among children and adolescents increased from 5842769.65 in 1990 to 6154651.76 in 2021, while the age-specific incidence rate decreased from 258.69 in 1990 to 233.50 in 2021, with a EAPC being −0.54. The 15–19 years group had the highest incidence and DALYs number, while 0–4 years group had the lowest. The projective model indicates that the burden for IOFBs will rise sharply in the next ten years.

**Conclusion:**

The global incidence and disease burden of blindness and vision loss in children and adolescents due to IOFB have shown a slight decline from 1990 to 2021. However, there may be a significant upward trend in the future, which requires the vigilant attention of policymakers.

## 1 Introduction

Ocular injuries are a leading cause of blindness, with an annual global incidence rate of 3.5/100,000 persons (1) and remained a significant public health issue worldwide (2). In particular, intraocular foreign bodies (IOFBs) stand out as a major source of morbidity among young individuals, comprising 6 to 42.1% of all open globe injuries (OGIs) (3–10). Prompt diagnosis and treatment are imperative to avert blindness or loss of the eye (11). Typically metallic, IOFBs often result from activities like hammering, drilling, gunfire, or explosions (8), and can affect any part of the eye, including both anterior and posterior segments. Due to their sharp nature and high velocity, they frequently become lodged in the posterior segment, with approximately two-thirds of IOFBs reported in this region (4).

Trauma associated with IOFBs demands meticulous investigation and swift intervention (12). Prompt recognition and appropriate management are crucial to prevent complications and ensure the best possible visual outcome (13). The prognosis for vision depends on various factors (3, 14, 15), including the size and location of the foreign body, its composition, the inflammatory response, the extent of tissue damage, the duration of injury, and any associated endophthalmitis or retinal detachment (16).

The Global Burden of Disease (GBD) study examined trends from 1990 to 2021, assessing the mortality and disability caused by hundreds of diseases, injuries, and risk factors around the world. The study encompassed 204 countries and territories, 288 causes of death, 371 diseases, injuries, and 88 risk factors. Previous studies have primarily focused on individuals across all age groups, with a predominant emphasis on adults (17, 18). There is a paucity of information regarding IOFBs in children. However, according to the data from the GBD 2021, foreign body injuries account for a significant proportion of eye injuries among children (19), with boys being more commonly affected than girls. With children being more prone to such injuries due to their natural curiosity and exploratory behavior. This study aims to analyze global trends in incidence and disability-adjusted life years (DALYs) over a 30-year period for IOFB injuries among children and adolescents and predict potential future patterns using statistical modeling. The findings can provide key information for researchers and to guide health policies and health service approaches.

## 2 Methods

### 2.1 Data collection

The GBD database is accessible through the IHME’s health data platform (https://ghdx.healthdata.org/gbd-2021). The data can be filtered based on various parameters such as cause (e.g., cataract, age-related macular degeneration), measure, location, age, sex, and year, ranging from 1990 to 2021. We downloaded the data on the number of incidence and DALYs, age-specific incidence rates, and DALYs rates from 1990 to 2021 by sex, age and SDI regions globally. The percentage calculation is defined as number of cases/total number of cases. Unintentional tissue damage from extraneous materials or substance in the orbital structure or eye is defined as IOFB, including ICD-9: 360.5–360.69, 374.86, 376.6, E914-E914.09; ICD-10: H02.81-H02.819, H44.6-H44.799. The World Health Organization (WHO) defines an adolescent as any person between ages 10 and 19 years. In this study, children and adolescents are defined as individuals aged 0 to 19 years old. Age groups were defined with 5-year intervals (20), specifically divided into 0–4 years, 5–9 years, 10–14 years, and 15–19 years.

DALYs are a key metric used in public health to quantify the overall disease burden from various health conditions or injuries. It’s equal to the years lived with disability (YLLs) plus the years of life lost (YLDs). Since YLD for IOFBs is zero, we have focused our analysis solely on DALYs. The estimated annual percent change (EAPC) is a statistical measure that projects the rate at which a particular variable is expected to change over time, typically on an annual basis. The formula and methods for calculating EAPC were described in detail previously (18). An EAPC value >0 indicates an increase over time, and an EAPC value <0 indicates a decrease over time. An EAPC value with a 95% CI of 0 indicates stability. The Socio-demographic Index (SDI) represents a summary of a location’s socio-economic development and is calculated based on a combination of variables that reflect the overall socio-economic status of a population. The SDI is scaled from 0 to 1, with higher values indicating better socio-economic conditions. According to SDI, the regions were divided into 5 grades: low SDI (< 0.46), low-middle SDI (0.46 ~ 0.61), middle SDI (0.61–0.71), high-middle SDI (0.71 ~ 0.81) and high SDI (> 0.81). Previous studies described the detailed methodology of GBD study (21). The GBD study data followed the guidelines for Accurate and Transparent Health Estimation Reporting for Population Health Research (GATHER).

### 2.2 Prediction model

Bayesian Age-Period-Cohort (BAPC, version 0.0.36) is a statistical model used for analyzing and predicting trends in disease incidence and mortality rates. It is based on the traditional Age-Period-Cohort (APC) model and incorporates integrated nested Laplace approximations (INLA) to address collinearity issues in parameter estimation. The projected population data was from the GBD website (https://ghdx.healthdata.org/record/ihme-data/global-population-forecasts-2017-2100). BAPC model was used to show the trends change in four age groups of male and female in the next ten years. The results were verified by the Nordpred (version 1.1) model.

### 2.3 Statistical analysis

The GBD database was used to extract data on the occurrence of IOFBs. Firstly, we have summarized and calculated the number of incidences, DALYs, and EAPC by sex, age, and SDI region globally. Secondly, we presented the difference between different SDI regions and correlation. Thirdly, we presented the trends in the number and rate of incidences and DALYs by age groups and sex from 1990 to 2021 globally. Finally, we utilized the BAPC model to forecast the incidence and DALYs for the next decade.

Statistical analyses and figures were depicted by R (version 4.3.2) and Rstudio. Data input and display were using GraphPad Prism (version 9.0.0). A *p* value <0.05 was considered statistically significant.

## 3 Results

### 3.1 Global burden of IOFBs

Globally, it is estimated that the incidence number of IOFBs among children and adolescents aged 0–19 years increased from 5842769.65 (95%UI: 3483076.85 to 9288322.81) in 1990 to 6154651.76 (95%UI: 3664992.73 to 9616079.1) in 2021, while the age-specific incidence rate decreased from 258.69 per 100 k (95%UI: 154.22 to 411.25) an 1990 to 233.5 per 100 k (95%CI: 139.04 to 364.82) in 2021, with a EAPC being −0.54 (95%CI: −0.72 to −0.35) (Table 1).

**Table 1:** The incidence and DALYs number and rate for IOFBs from 1990 to 2021 by sex and age group among children and adolescents globally.

	Incidence					DALYs				
	Rate, per 100,000 (95% UI)		Number (95%UI)		EAPC (95%CI)	Rate, per 100,000 (95% UI)		Number (95%UI)		EAPC (95%CI)
	1990	2021	1990	2021		1990	2021	1990	2021	
Global	258.69 (154.22 to 411.25)	233.5 (139.04 to 364.82)	5842769.65 (3483076.85 to 9288322.81)	6154651.76 (3664992.73 to 9616079.1)	−0.54 (−0.72 to −0.35)	2.21 (0.88 to 4.59)	1.96 (0.75 to 4)	49938.33 (19797.67 to 103604.22)	51706.67 (19819.61 to 105534.33)	−0.58 (−0.76 to −0.41)
**Sex**										
Male	335.91 (196.84 to 533.33)	299.73 (176.81 to 467.84)	3887772.59 (2278190.25 to 6172597.86)	4071825.24 (2402006.03 to 6355552.12)	−0.67 (−0.87 to −0.46)	2.84 (1.11 to 5.88)	2.49 (0.95 to 5.06)	32828.06 (12842.74 to 67998.91)	33797.9 (12862.97 to 68712.59)	−0.7 (−0.9 to −0.51)
Female	177.53 (104.36 to 285.44)	163.06 (96.36 to 255.55)	1954997.06 (1149192.41 to 3143223.33)	2082826.52 (1230809.19 to 3264228.07)	−0.3 (−0.46 to −0.14)	1.55 (0.64 to 3.15)	1.4 (0.55 to 2.88)	17110.28 (7002.53 to 34736.25)	17908.77 (7004.18 to 36812.82)	−0.36 (−0.51 to −0.2)
SDI quintiles										
High SDI	427.44 (269.21 to 649.12)	451 (280.67 to 682.72)	1074236.07 (676576.7 to 1631357.36)	1049582.44 (653194.15 to 1588835.59)	0 (−0.27 to 0.27)	3.69 (1.49 to 7.27)	3.89 (1.57 to 7.67)	9281.13 (3757.18 to 18268.16)	9048.12 (3657.11 to 17844.78)	0 (−0.27 to 0.27)
High-middle SDI	318.1 (183.4 to 507.92)	276.64 (164.43 to 428.72)	1177503.68 (678868.97 to 1880132.12)	839216.43 (498814.02 to 1300580.33)	−0.98 (−1.22 to −0.74)	2.69 (1.05 to 5.71)	2.28 (0.86 to 4.78)	9959.6 (3887.07 to 21154.53)	6918.36 (2600.61 to 14489.37)	−1.04 (−1.27 to −0.81)
Middle SDI	232.82 (126.33 to 390.55)	213.1 (121.57 to 349)	1779996.46 (965860.67 to 2985930.5)	1596507.11 (910799.35 to 2614641.1)	−0.8 (−1.15 to −0.45)	1.97 (0.75 to 4.33)	1.74 (0.64 to 3.82)	15033.15 (5711.16 to 33125.44)	13028.11 (4767.18 to 28601.44)	−0.89 (−1.22 to −0.56)
Low-middle SDI	219.95 (132.73 to 342.13)	205.17 (124.49 to 313.98)	1299933.4 (784463.12 to 2022098.28)	1568301.75 (951561.62 to 2400006.36)	−0.12 (−0.19 to −0.04)	1.9 (0.76 to 3.81)	1.74 (0.66 to 3.57)	11217.68 (4482.74 to 22518.1)	13275.86 (5020.53 to 27320.34)	−0.18 (−0.25 to −0.12)
Low SDI	181.63 (112.26 to 270.41)	187.88 (114.58 to 285.33)	507805.57 (313854.48 to 755996.39)	1097616.87 (669411.53 to 1666917.82)	0.09 (0.08 to 0.1)	1.58 (0.64 to 3.2)	1.61 (0.63 to 3.31)	4418.55 (1790.8 to 8959.84)	9407.18 (3669.27 to 19352.01)	0.04 (0.02 to 0.05)
Age group										
0–4, y	133.23 (74.36 to 247.41)	123.93 (70.32 to 213.09)	825966.15 (460982.74 to 1533796.5)	815665.34 (462835.42 to 1402510.35)	−0.3 (−0.4 to −0.21)	1.04 (0.35 to 2.43)	0.96 (0.32 to 2.23)	6443.15 (2153.54 to 15076.25)	6331.46 (2097.4 to 14665.96)	−0.32 (−0.41 to −0.23)
5–9, y	207.57 (95.97 to 387.11)	188.54 (88.93 to 342.8)	1211217.63 (560038.43 to 2258920.22)	1295391.74 (611011.43 to 2355241.55)	−0.43 (−0.57 to −0.28)	1.73 (0.59 to 4.02)	1.54 (0.52 to 3.55)	10070.03 (3454.02 to 23434.88)	10602.47 (3575.08 to 24388.87)	−0.47 (−0.6 to −0.33)
10–14, y	306.12 (158.13 to 521.72)	274.17 (145.07 to 456.86)	1639804.96 (847058.61 to 2794777.79)	1827701.79 (967101.73 to 3045612.64)	−0.56 (−0.76 to −0.35)	2.64 (0.97 to 5.52)	2.31 (0.83 to 4.8)	14120.17 (5170.97 to 29579.11)	15410.69 (5555.56 to 32001.04)	−0.6 (−0.8 to −0.41)
15–19, y	416.96 (193.63 to 776.06)	355.12 (168.03 to 622.81)	2165780.9 (1005749.11 to 4031040.19)	2215892.89 (1048442.63 to 3886211.69)	−0.77 (−1.04 to −0.5)	3.72 (1.34 to 8.55)	3.1 (1.12 to 7.27)	19304.99 (6984.24 to 44389.23)	19362.05 (7007.04 to 45333.69)	−0.81 (−1.07 to −0.55)

Moreover, the age-specific incidence rate of number from 1990 to 2021 showed downward trends, with EAPC of −0.67 (95%CI: −0.87 to −0.46) for male, and − 0.3 (95%CI: −0.46 to −0.14) for female, respectively (Table 1).

**Figure 1:**
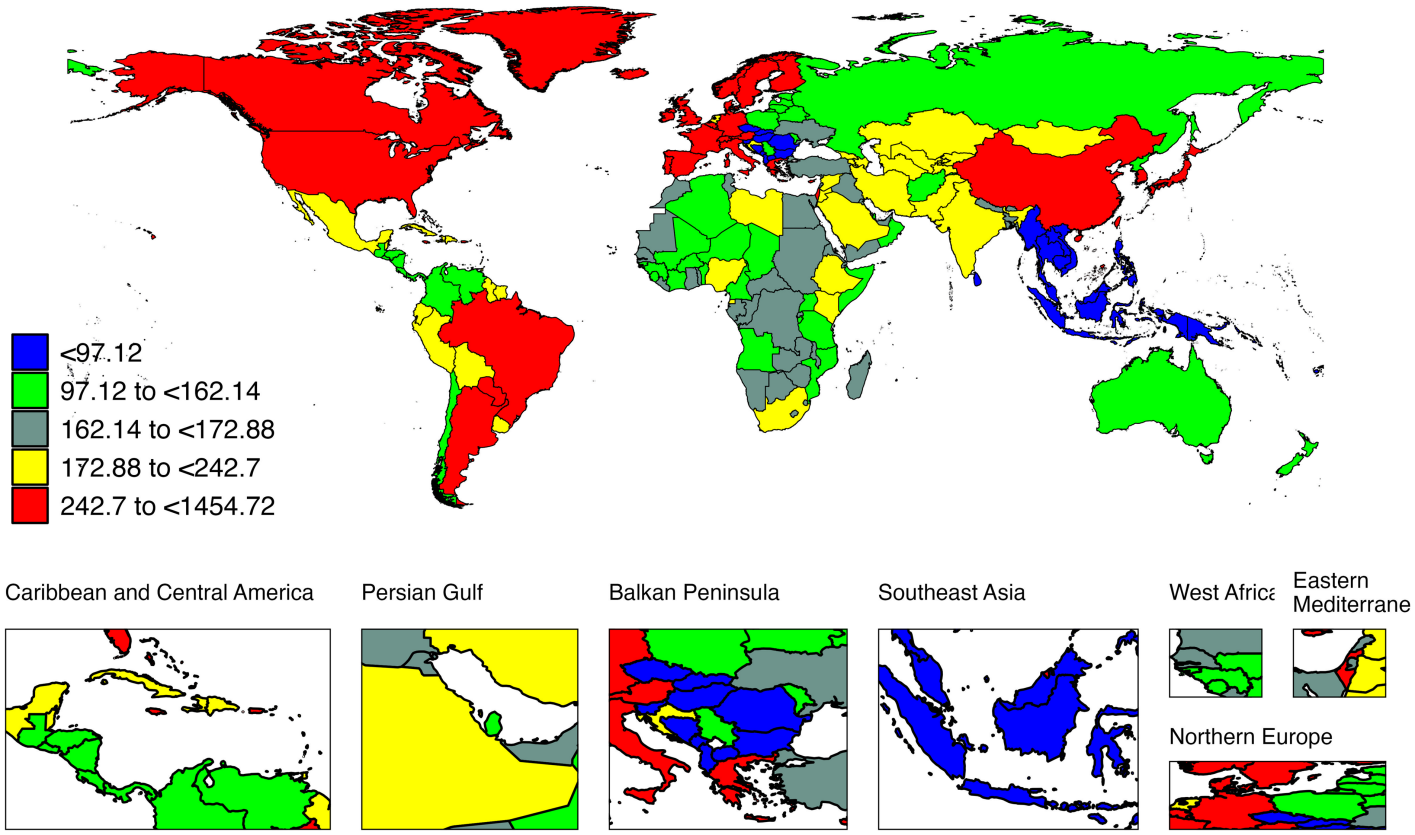
The age-specific rate of incidence of IOFBs among children and adolescents for both sexes in 204 countries and territories in 2021.

At national levels, the highest age-specific incidence rate was seen in Republic of Italy (1454.72 per 100,000, 95% UI: 961.06 to 2022.56) (Figure 1), while the lowest age-specific incidence rate worldwide was noted in Czech Republic (44.93 per 100 k, 95% UI: 22.91 to 75.12) in 2021 (Supplementary Figure 1). Republic of Italy had the highest incidence rate decrease, with an EAPC of −1.98. Philippines had the highest incidence rate increase, with an EAPC of 0.91.

### 3.2 SDI level

**Figure 2:**
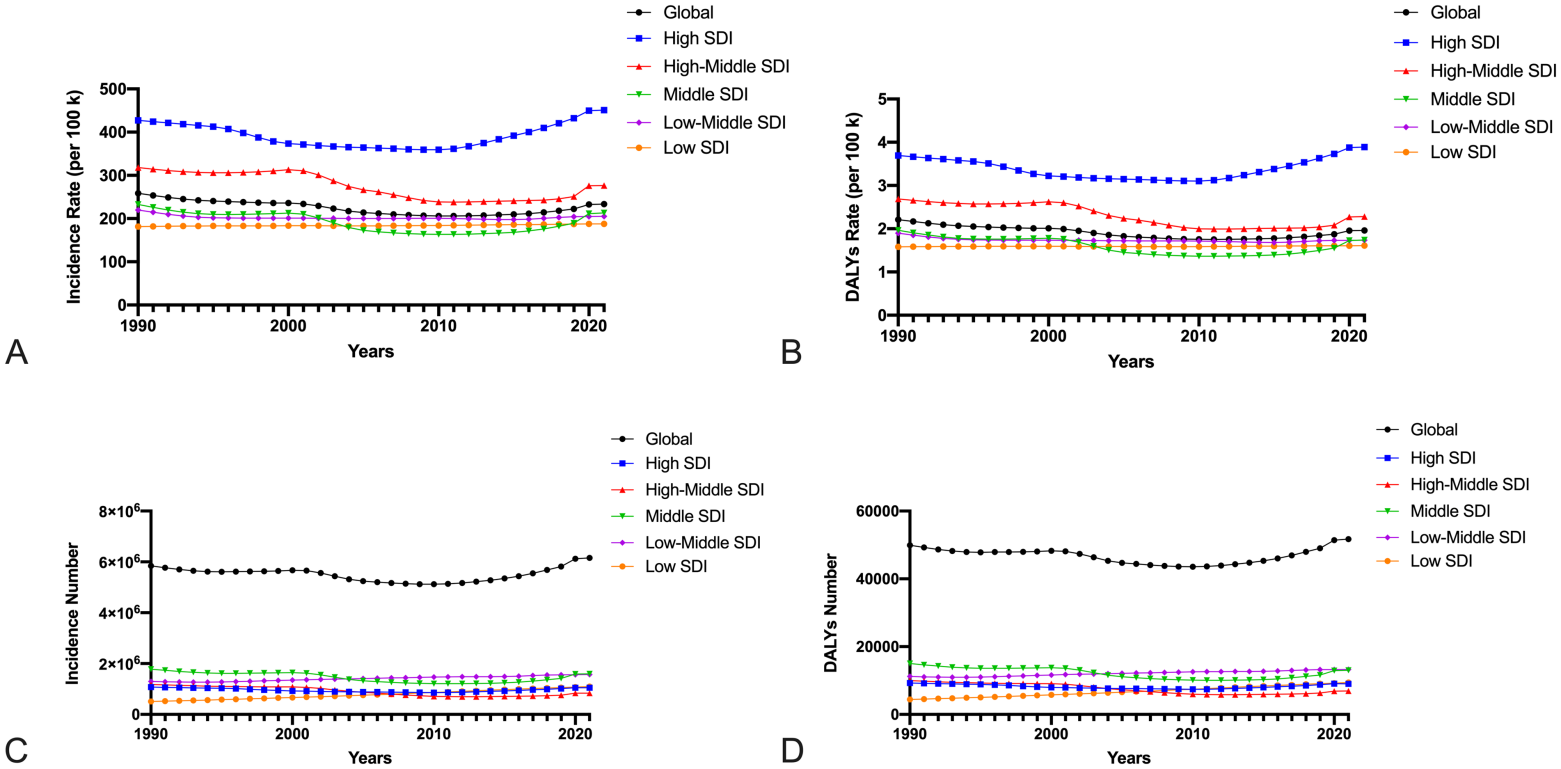
The incidence **(A,C)** and DALYs **(B,D)** rate and number of IOFBs among children and adolescents from 1990 to 2021 globally by SDI region.

**Figure 3:**
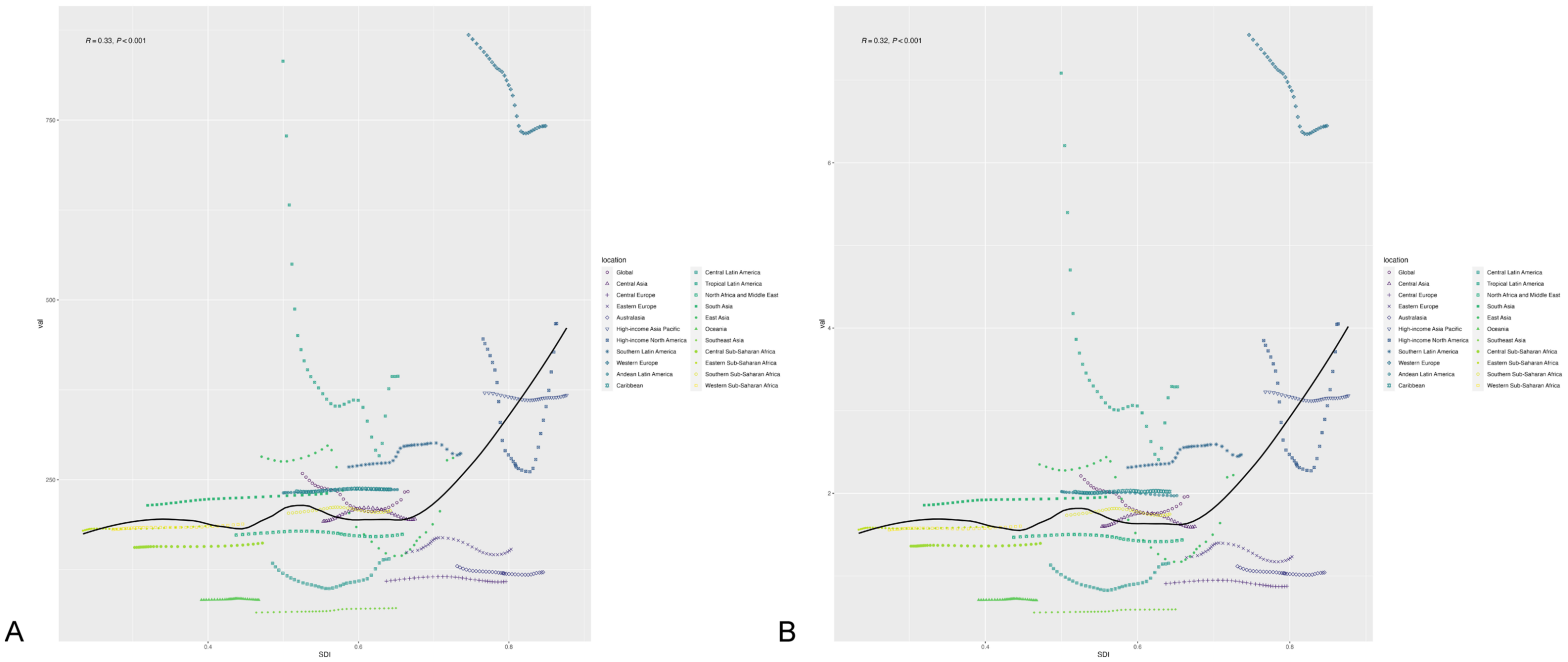
**(A)** The change trends and correlation analyses of age-specific incidence rate and SDI from 1990 to 2021 in 21 regions. **(B)** The change trends and correlation analyses of age-specific DALY rate and SDI from 1990 to 2021 in 21 regions.

Among the five SDI region, we observed a slight upward trend in regions with low SDI region, while all other regions demonstrated a decreasing trend. Additionally, regions with high SDI had the highest incidence and DALYs rate from 1990 to 2021(Figures 2A,B). The rate of incidence and DALYs for high SDI and high-middle SDI regions are higher than the global average. On the contrary, the number of incidence and DALYs for middle and low-middle SDI regions have the most among five SDI regions (Figures 2C,D). The middle SDI region has the highest percent of all regions, declining from 30.46% in 1990 to 25.48% in 2021. In 1990, the proportion of low SDI region was the lowest, only 8.69%, and in 2021, the proportion of high SDI region was the lowest, 17.05% (Supplementary Figure 2). In addition, correlation analysis found that as the SDI increased, their corresponding age-specific incidence and DALY rate would show an upward trend (*R* = 0.33, 0.32; *p* < 0.001) (Figures 3A,B).

### 3.3 Time trends of incidence and DALYs of IOFBs from 1990 to 2021 by sex

**Figure 4:**
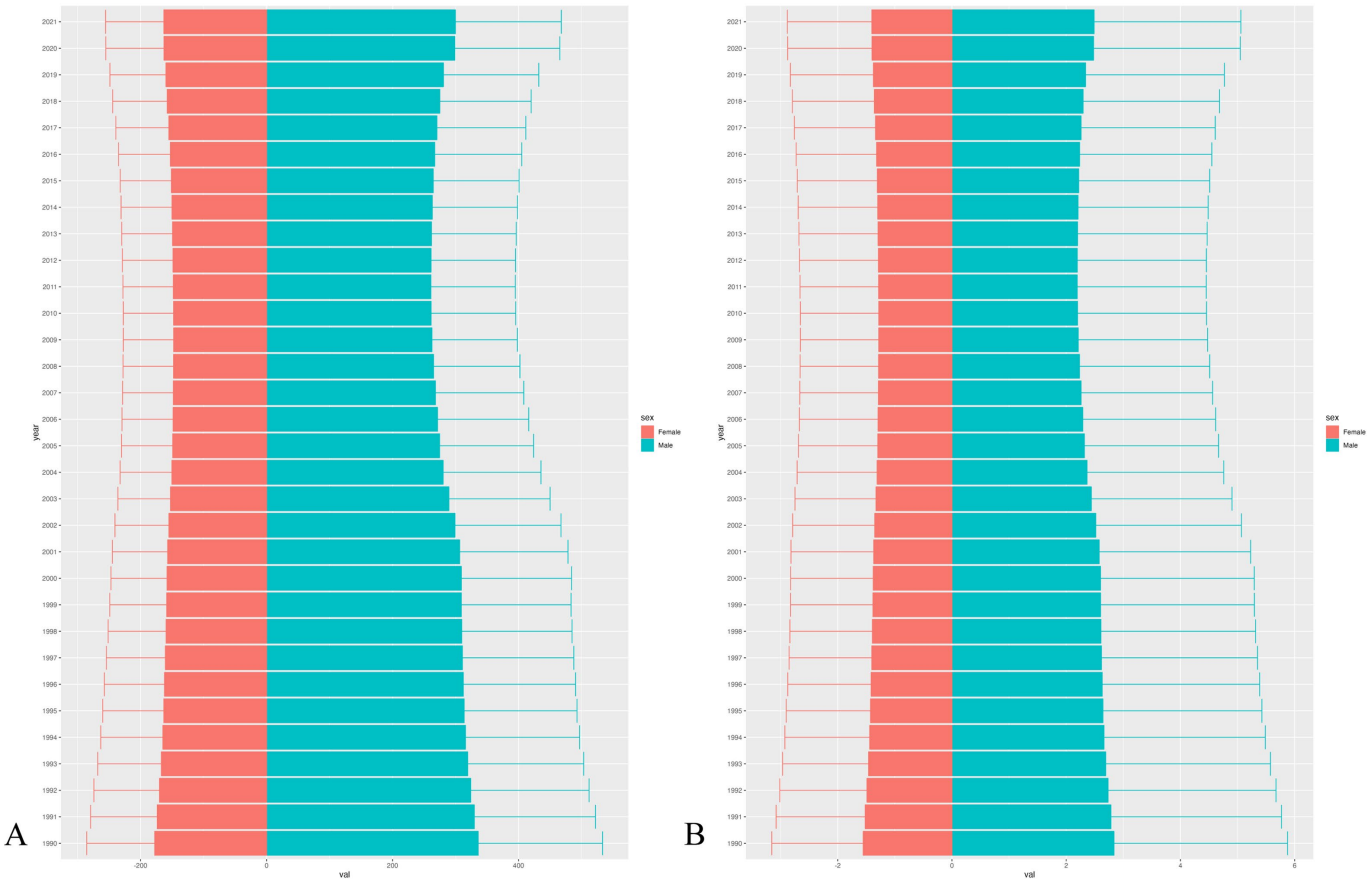
The incidence **(A)** and DALYs rate **(B)** of IOFBs among children and adolescents from 1990 to 2021 by sex.

As shown in Figure 4, we observed that the age-specific incidence rates for males have gradually declined from 1990 (335.91 per 100 k, 95% UI: 196.84 to 533.33), reaching the lower point in 2011 (260.82 per 100 k, 95% UI: 162.07 to 394.86) and began to rise gradually until 2021 (299.73 per 100 k, 95% UI: 176.81 to 467.84) with an EAPC of −0.67 (95% UI: 0.87 to −0.46). We also found that the incidence and DALY rates of males was significantly higher than that of females. On the other hand, the incidence numbers for females have gradually declined from 1954997.06 (95% UI: 1149192.41 to 3143223.33) in 1990, reaching the lower point in 2007(1764135.01, 95% UI: 1065086.07 to 2724809.54) and began to rise gradually to 2082826.52(95% UI: 1230809.19 to 3264228.07) in 2021(Supplementary Figure 3).

### 3.4 Time trends of incidence and DALYs of IOFBs from 1990 to 2021 by age

**Figure 5:**
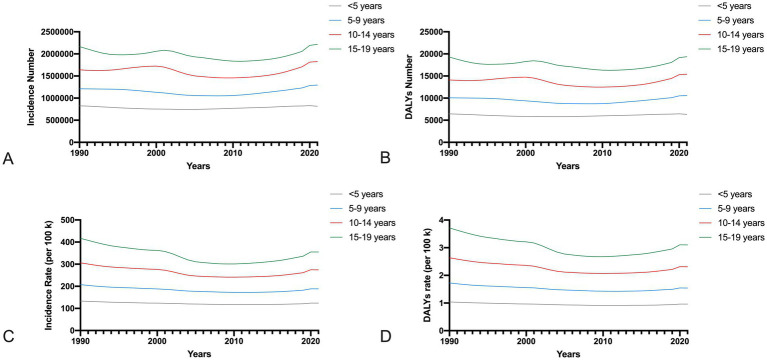
The incidence and DALYs number **(A,B)** and rate **(C,D)** of IOFBs among children and adolescents in 1990 and 2021 by age group.

The trend in IOFBs differed according to age. Figure 5 gives a brief view of age-specific disease burden of IOFBs globally from 1990 to 2021. Among people aged 0–19 years, adolescents aged 15–19 years had the highest incidence and DALYs rate and number of IOFBs, while children aged 0–4 years had the lowest. We can observe that as age increases, the burden of disease also progressively rises.

It is estimated that the age-specific incidence rate of IOFBs aged 0–4 years decreased from 133.23 per 100 k (95%UI: 74.36 to 247.41) in 1990 to 123.93 per 100 k (95%UI: 70.32 to 213.09) in 2021. The age-specific incidence rate aged 15-19 years declined most dramatically from 416.96 per 100 k (95%UI: 193.63 to 776.06) in 1990 to 355.12 per 100 k (95%UI: 168.03 to 622.814) in 2021, with an EAPC of −0.77.

### 3.5 Future trend in the next ten years

**Figure 6:**
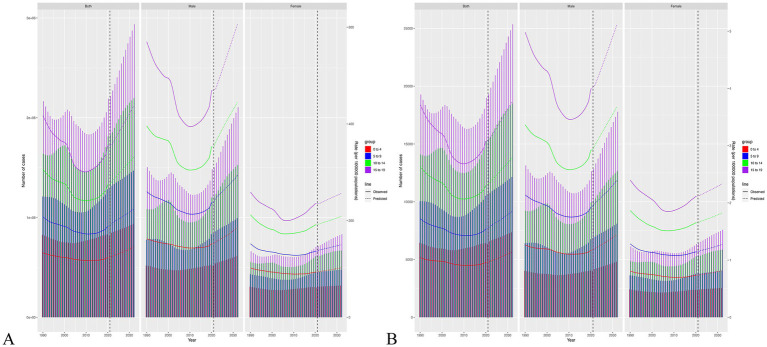
The change trends of the incidence **(A)** and DALYs **(B)** numbers and rate from 1990 to 2032 globally by sex and age.

The BAPC models project a significant increase in the incidence and DALYs trends from 2022 onwards to 2032. An upward trend was observed in both men and women, with males being more pronounced (Figure 6). The projection model shows that by 2032, the age-specific incidence rate for IOFBs aged 15–19 years will rise to 606.19 per 100 k for men and 256.20 per 100 k for women. In 2032, the age-specific incidence rate of IOFBs between the ages of 0–4 years will rise to 185.60 per 100 k for men and 102.38 per 100 k for women. Notably, the 15–19 years age group had the highest age-specific DALYs rate (5.12 per 100 k for male, 2.33 per 100 k for female) in 2032. In contrast, the 0–4 years age group had the lowest age-specific DALYs rate (1.45 per 100 k for male, 0.82 per 100 k for female). The Norpred model also found an upward trend in the next ten years (Supplementary Figures 4, 5).

## 4 Discussion

In this study, we reported the disease burden of IOFBs from 1990 to 2021 globally, stratified by age, sex and SDI region, through a secondary analysis of the GBD 2021 database. Our findings can assist policymakers in assessing and optimizing current eye health services. We observed a slight decline in the incidence rate of IOFBs from 1990 to 2021, accompanied by an increase in the number of cases, a result that may be related to population growth. As the global population increases, the total number of people with IOFBs could rise even if the incidence rate remains stable or declines. The current disease burden remains high, particularly among boys aged 15–19 years. Additionally, predictive models suggest a significant increase in the disease burden over the next decade, which also warrants public attention. Some clinical studies focus on the incidence and risk factors in regional or smaller-scale studies (8, 22, 23), or look at whole populations (18), while our study targets the incidence and DALYs of children and adolescents globally. This provides more comprehensive and targeted epidemiological data for IOFBs related to children and adolescents.

Interestingly, the results showed that regions with high SDI had the highest incidence and DALYs rate from 1990 to 2021, which may be closely related to socioeconomic development, cognitive differences, biases in data reporting and the development and progress of medical diagnostic technology. First, in the high SDI region, there may be a greater number of medical institutions and professionals involved in the collection and reporting of data, which could lead to more comprehensive and accurate reporting of eye injury cases in these regions. Second, schools and parents may pay more attention to eye injuries in children, being more capable of identifying affected patients in the high SDI region. Third, in regions with a higher degree of urbanization, children may have easier access to high-speed traffic, construction sites, and other industrial environments, all of which are potential risk factors for eye injuries.

Globally, it is not uncommon for children to experience IOFBs. Identifying the risk factors for IOFBs is crucial for reducing the incidence. Research (23) has indicated that playgrounds and schools are common settings for student eye injuries. Accidental punctures from sharp objects such as wood, metal bodies, glass, and pencils during play, as well as injuries from firecrackers, and damages caused by traffic accidents. Danger is everywhere. Children have a weaker ability to discern danger, hence it is imperative for parents and teachers to provide appropriate education. Children should be prohibited from approaching hazardous objects and engaging in risky activities. Moreover, safety education and protective measures should be tailored to the age and sex of the child to ensure their effectiveness. A study revealed that plant branches were the most common cause of injuries in females while fireworks were more prevalent among males (5). Clinical characteristics of pediatric IOFBs injuries differ from those in adults. Peyman A et al. found boys had a greater IOFB rate but lower blindness odds than girls (24) while a retrospective analysis of 484 pediatric inpatients did not find significant sex differences in IOFBs (5). However, our study discovered that both the incidence rate and DALYs rate were significantly lower among girls compared to boys which may be attributed to several factors: Firstly, boys tend to spend more time participating in outdoor activities or occupations where foreign bodies like dust particles, debris or insects are more prevalent, thus increasing their exposure risk toward eye injuries. Secondly, boys might have a greater inclination toward playing with toys or engaging in hobbies involving small parts or sharp objects such as building models or using tools which further elevates their risk of eye injury occurrence. Thirdly, during childhood and adolescence period especially, boys, tend to engage in more risk-taking behaviors, such as adventurous play or participation in contact sports, which may increase the likelihood of eye injuries.

Meanwhile, we observed that the burden of IOFBs predominantly affects individuals in the 15 to 19 age group, irrespective of sex. Even after adjusting for population size, the rate of DALYs remains notably high within the 15 to 19 age group, underscoring an age-related escalation in burden. We hypothesize that age could serve as a risk factor for IOFB onset. This is similar to the results of a previous study (22). This may be attributed to the heightened curiosity and proactive engagement with hazardous objects typically seen in adolescents aged 15–19, contrasting with the more constrained and supervised environment of children aged 0–4. Our findings underscore the importance of tailored policies targeting distinct age groups to mitigate the incidence and disease burden associated with IOFBs.

Our study reveals a significant reduction in the disease burden from 1990 to 2021. However, the burden of IOFBs has been increasing sharply since 2011 and our BAPC predictive model also forecasts a notable increase in the incidence and DALY rates of IOFBs among the pediatric population aged 0–19 years post-2020, affecting both males and females equally. Several factors could be driving this upward trend: First, with economic development, the market has seen an emergence of many hazardous toys, escalating the risks. Second, the progression of urbanization and industrialization exposes children to environments such as industrial materials and construction sites, which could further increase the danger. Third, as children engage in more outdoor activities and sports, their exposure to potential hazardous objects may also rise. Finally, it may be that previous types of IOFB were limited, but later the potential sources of IOFB increased, leading to a predicted rise. On the other hand, model predictions have uncertainties and confounding factors, such as technological advances or changes in safety regulations, so the application of forecast results needs to be treated more accurately. Given the continuous increase in ocular trauma, the disease burden it causes could become one of the major public health issues in China in the near future. To address this preventable and avoidable condition (25), it is crucial to raise public awareness, restrict children’s access to fireworks (10), advocate for the use of protective eyewear during sports, and implement public policies that prioritize the early diagnosis and treatment of ocular foreign body injuries. The insights from our research offer a strategic approach to mitigate the escalating disease burden associated with IOFBs and reduce their impact on society.

Despite our efforts to provide a comprehensive analysis of the trends of IOFB, the data collection originated from various countries, which may have inherent inconsistencies in collection methods that could impact the results. Additionally, our study did not delve into the potential risk factors correlated with IOFBs, primarily because such data is absent within the GBD database. This gap in information hinders our ability to explore the etiology of IOFBs more comprehensively. Future research should focus on identifying possible risk factors to fundamentally prevent the disease’s harm to children.

## 5 Conclusion

In summary, the incidence and disease burden of blindness and vision loss in children and adolescents due to IOFBs have shown a decline from 1990 to 2021. However, there may be a significant upward trend in the next decade, which requires the vigilant attention of policymakers. Additionally, we found that being male and older may be associated with a greater burden. We hope that this study can provide data to support policymakers in prioritizing investing in more effective interventions to reduce the chances of injury and improving health care systems to reduce IOFBs among children and adolescents due to different causes in the future.

## Data Availability

The original contributions presented in the study are included in the article/Supplementary material, further inquiries can be directed to the corresponding authors.
