# Supplementary material for: The trends and future projections of intraocular foreign bodies among children and adolescents: a global analysis
Source: Front Med (Lausanne). 2025 Feb 13;12:1512959. doi: 10.3389/fmed.2025.1512959 (PMC11866634; doi:10.3389/fmed.2025.1512959)
Supplement: Supplementary file 1 [file Data_Sheet_1.docx]

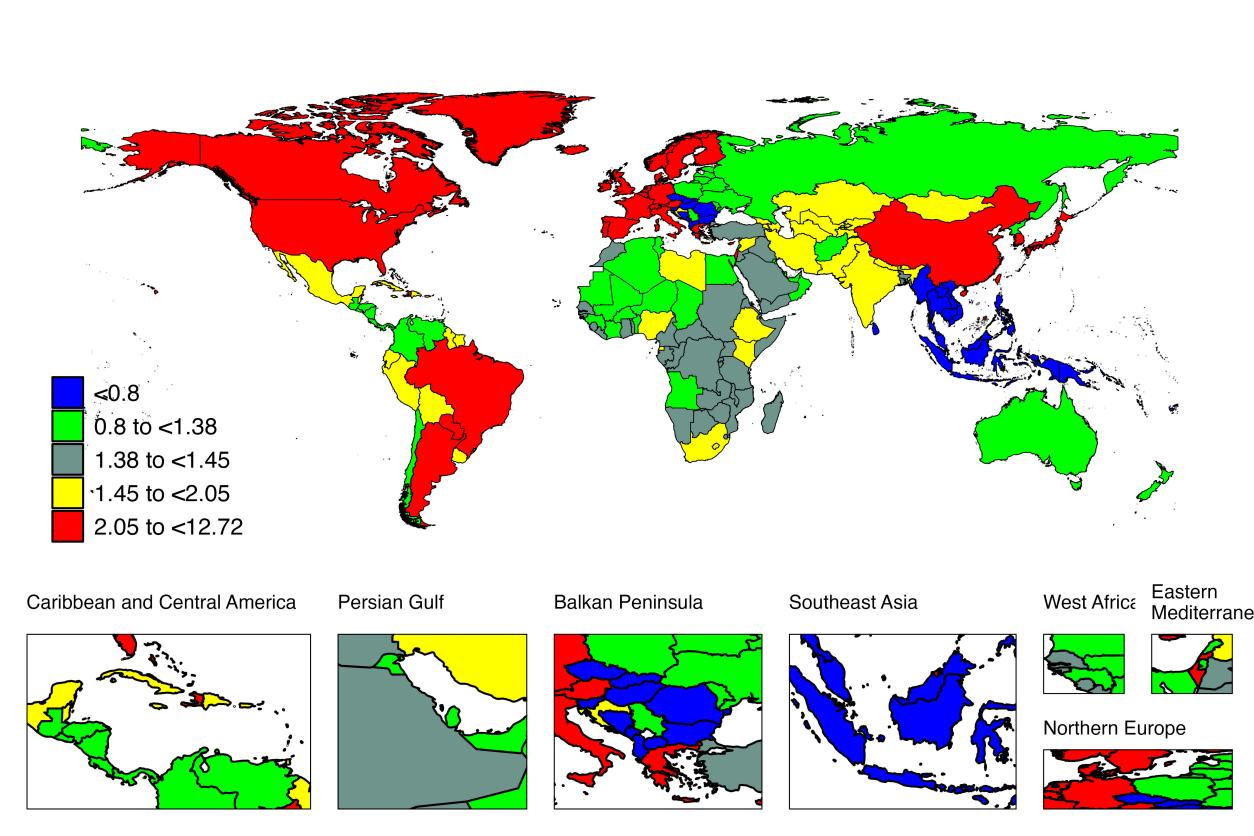


Supplementary Figure 1 The age-specific rate of DALYs of IOFBs among children and adolescents for both sexes in 204 countries and territories in 2021.


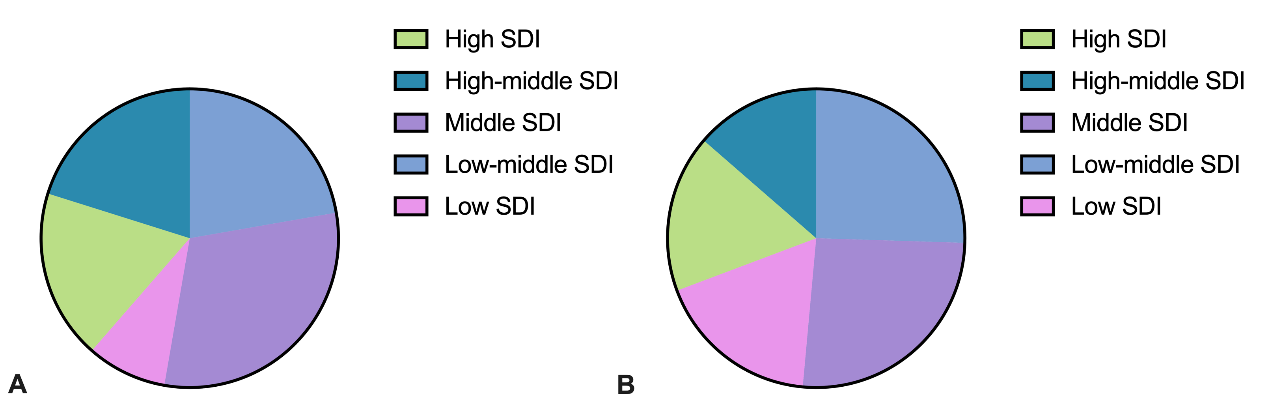


Supplementary Figure 2 The percent of age-specific incidence rate in IOFBs among children and adolescents by SDI regions in 1990 (A) and 2021 (B).


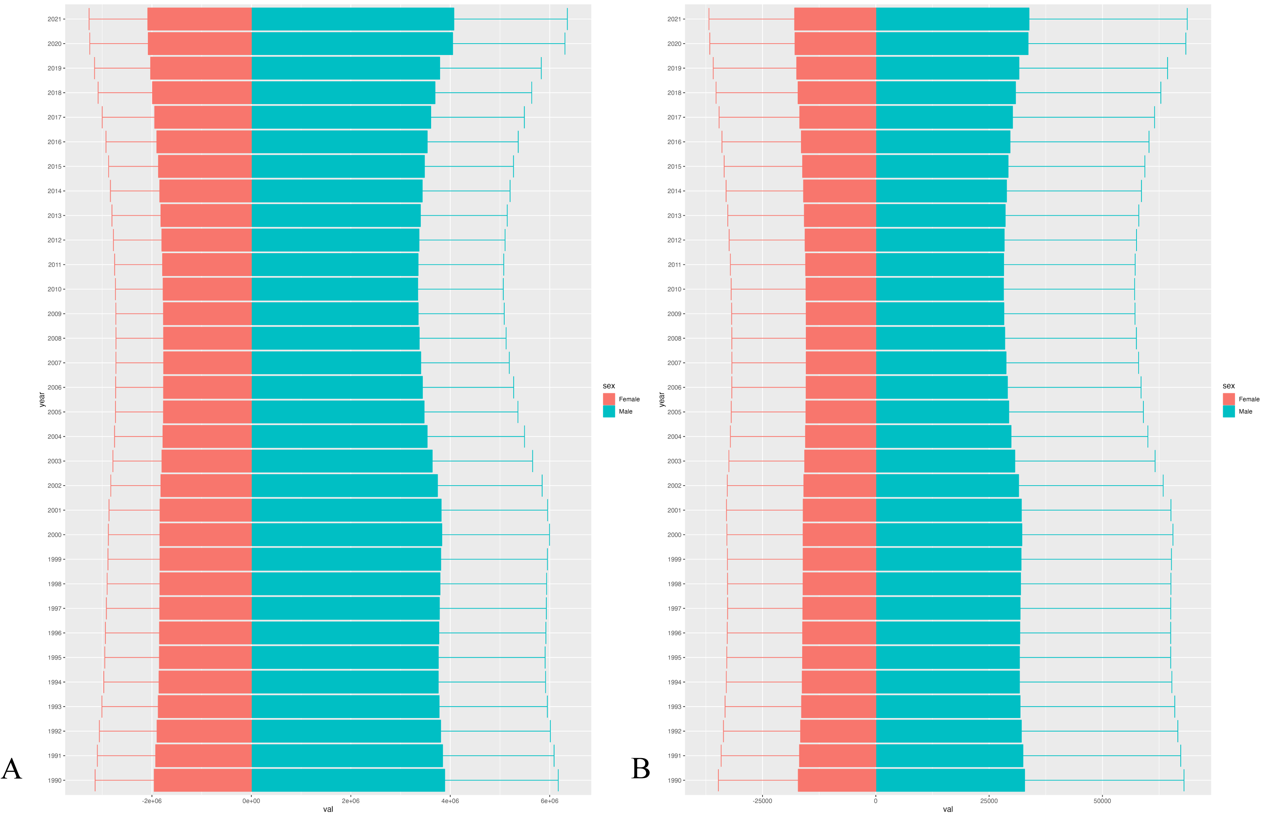


Supplementary Figure 3 The incidence (A) and DALYs number (B) of IOFBs from 1990 to 2021 by sex.


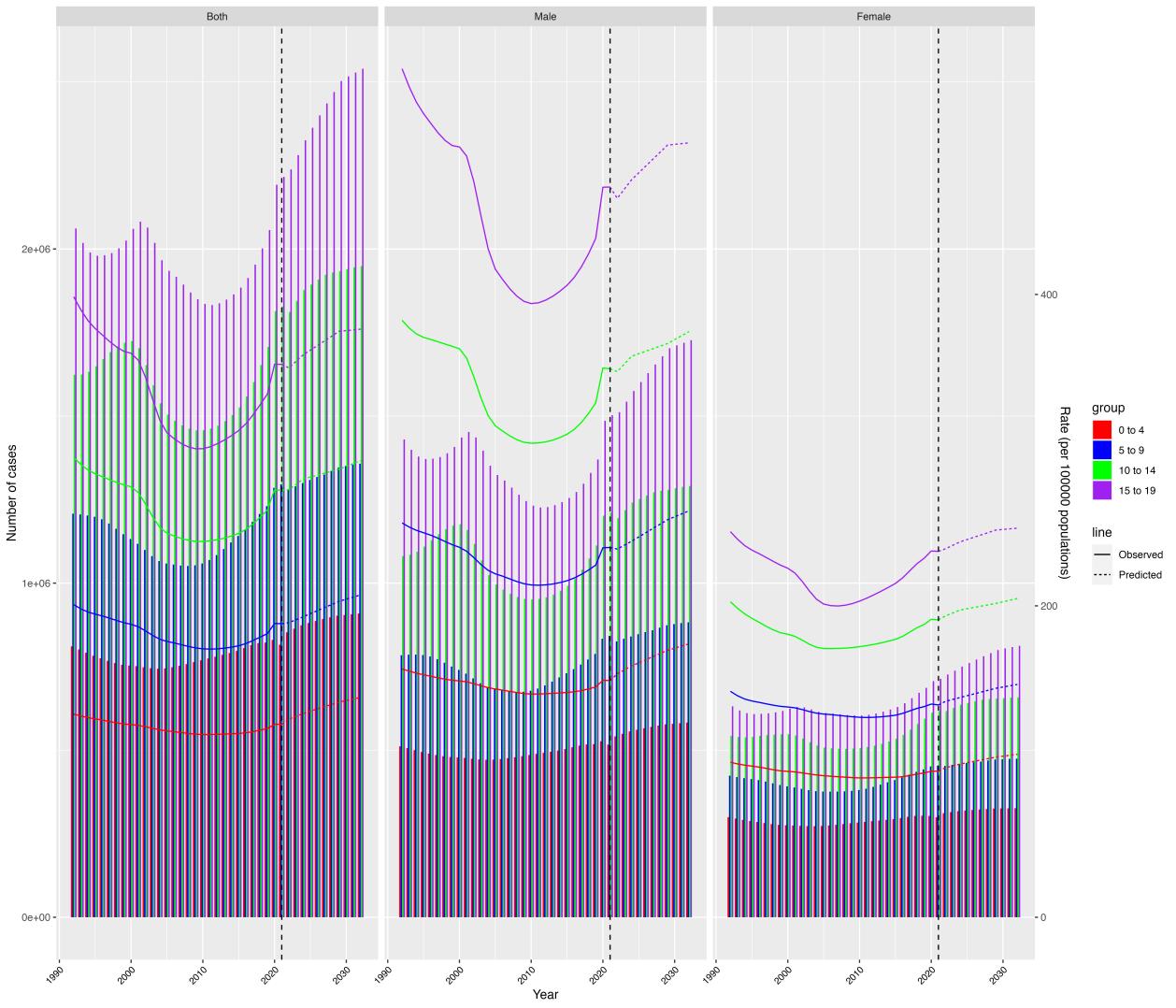


Supplementary Figure 4 The change trends of the incidence numbers and rate from 1992 to 2032 globally by sex and age.


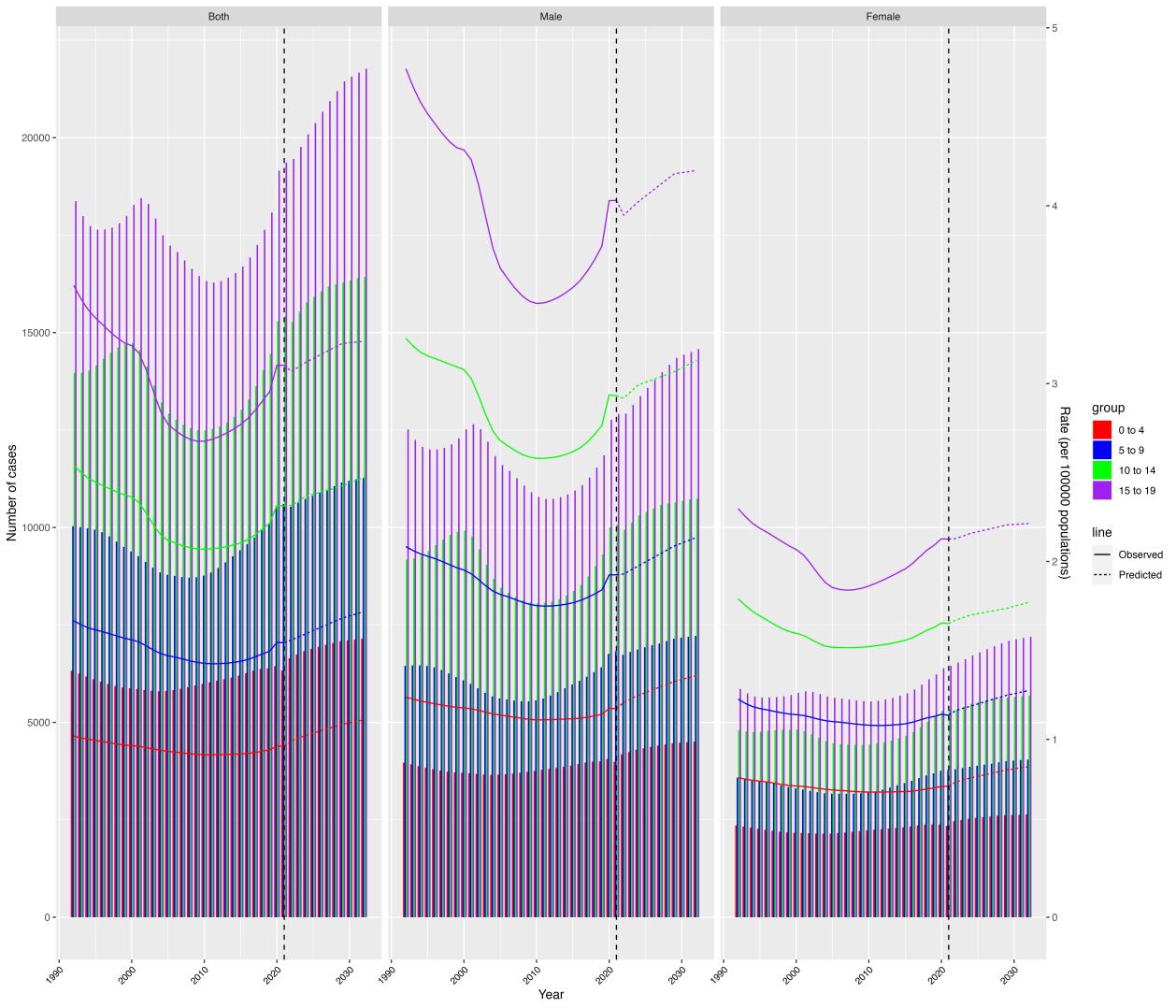


Supplementary Figure 5 The change trends of the DALYs number and rate from 1992 to 2032 globally by sex and age.
